# Optimization of Proton CT Detector System and Image Reconstruction Algorithm for On-Line Proton Therapy

**DOI:** 10.1371/journal.pone.0156226

**Published:** 2016-05-31

**Authors:** Chae Young Lee, Hankyeol Song, Chan Woo Park, Yong Hyun Chung, Jin Sung Kim, Justin C. Park

**Affiliations:** 1 Department of Radiological Science, Yonsei University, Wonju, Republic of Korea; 2 Department of Radiation Oncology, Yonsei Cancer Center, Yonsei University College of Medicine, Seoul, Republic of Korea; 3 Department of Radiation Oncology, University of Florida, Gainesville, Florida, United States of America; Chongqing University, CHINA

## Abstract

The purposes of this study were to optimize a proton computed tomography system (pCT) for proton range verification and to confirm the pCT image reconstruction algorithm based on projection images generated with optimized parameters. For this purpose, we developed a new pCT scanner using the Geometry and Tracking (GEANT) 4.9.6 simulation toolkit. GEANT4 simulations were performed to optimize the geometric parameters representing the detector thickness and the distance between the detectors for pCT. The system consisted of four silicon strip detectors for particle tracking and a calorimeter to measure the residual energies of the individual protons. The optimized pCT system design was then adjusted to ensure that the solution to a CS-based convex optimization problem would converge to yield the desired pCT images after a reasonable number of iterative corrections. In particular, we used a total variation-based formulation that has been useful in exploiting prior knowledge about the minimal variations of proton attenuation characteristics in the human body. Examinations performed using our CS algorithm showed that high-quality pCT images could be reconstructed using sets of 72 projections within 20 iterations and without any streaks or noise, which can be caused by under-sampling and proton starvation. Moreover, the images yielded by this CS algorithm were found to be of higher quality than those obtained using other reconstruction algorithms. The optimized pCT scanner system demonstrated the potential to perform high-quality pCT during on-line image-guided proton therapy, without increasing the imaging dose, by applying our CS based proton CT reconstruction algorithm. Further, we make our optimized detector system and CS-based proton CT reconstruction algorithm potentially useful in on-line proton therapy.

## Introduction

Proton therapy can deliver high doses to well-defined tumor volumes without damaging critical normal tissue due to the Bragg peak. For successful treatment, the relative stopping power (RSP) distributions inside patients’ bodies must be determined. Currently, stopping power calculations are performed using X-ray computed tomography (xCT), and patients are positioned by employing X-ray radiographs [1–2].

However, xCT calculations are intrinsically limited owing to the fundamental differences between the physical interactions of photons and protons, and xCT had been shown to yield 2–3 mm errors in estimated proton ranges [3]. In order to overcome this limitation, proton computed tomography (pCT), which uses protons directly for imaging, has been developed for high-precision planning. pCT has the potential to significantly improve the accuracy of proton therapy treatment planning and the alignment of target volumes with proton beams. Thus, pCT could reduce the uncertainties in measurements of materials’ relative proton stopping powers compared to those obtained using xCT images. It is also useful for pretreatment verification of patients’ positions relative to proton beams. Another advantage of pCT is that it could be further developed to employ imaging doses lower than those employed in xCT [4]. Furthermore, pCT can accurately construct RSP maps in phantoms. The main drawback of pCT is its low spatial resolution compared to that of xCT, which is related to multiple Coulomb scattering (MCS). Owing to MCS, protons passing through matter undergo multiple small-angle deflections and lose energy [5–6]. Research groups have performed investigations related to this problem during the past few decades.

In this paper, we propose an optimized pCT scanner system. To develop this system, the Geometry and Tracking (GEANT) 4.9.6 simulation software was employed. First, a GEANT4 simulation study was performed to optimize the system geometry for pCT. Then, using the GEANT4 simulation results, tomographic images were obtained via standard filtered back projection, the simultaneous algebraic reconstruction technique (SART), and iterative image reconstructed with compressed sensing (CS) regularization. Herein, we discuss and evaluate pCT images obtained using the three aforementioned reconstruction methods and demonstrate that superior spatial resolutions can be achieved in pCT images acquired by using CS reconstruction.

## Materials and Methods

### Physical principle of pCT

A pCT system provides precise values of the protons’ entrance and exit energies, locations, and directions. As described by the Bethe–Bloch equation, protons primarily lose energy through inelastic collisions with atomic electrons (atomic excitation and ionization) in matter [7]. The proton energy loss per unit track length, d*E*/d*x*, which is mainly caused by atomic excitation and ionization, can be expressed as follows:

$$-\frac{dE}{dx}\left(r\right)=\eta_{e}\left(r\right)F\left(I\left(r\right),E\left(r\right)\right)$$ (1)

Here, *η*_e_(*r*) is the electron density relative to water, *r* is the spatial location, *I*(*r*) is the mean ionization potential of the medium, *E*(*r*) is the proton energy, and *F* is a known function of *I* and *E* that is defined by the Bethe–Bloch equation as follows:

$$F\left(I\left(r\right),E\left(r\right)\right)=K\frac{1}{\beta^{2}\left(E\right)}\left[\text{ln}\left(\frac{2m_{e}c^{2}}{I\left(r\right)}\frac{\beta^{2}\left(E\right)}{1-\beta^{2}\left(E\right)}\right)-\beta^{2}\left(E\right)\right]$$ (2)

The constant K = 0.17 MeV/cm combines various physical factors, *m*_e_ is the electron mass, *c* is the speed of light, and *β*(*E*) is the proton velocity relative to *c*. Using Eq 2, the RSP of a phantom can be determined:

$$\int_{L}\eta_{e}\left(r\right)dx=\int_{E_{in}}^{E_{out}}\frac{dE}{S_{water}\left(E\right)}$$ (3)

where *L* is the estimated proton path, *E*_in_ is the proton entry energy, and *E*_out_ is the proton exit energy. As described above, pCT systems can perform reconstructions by directly using proton RSP values.

### pCT system configuration

This study was performed using the GEANT 4.9.6 simulation toolkit, which is often used the passage of particles through matter [8]. The GEANT4 simulations used the material specifications obtained from the National Institute of Standards and Technology (NIST) [9].

According to the NIST PSTAR (Stopping Power and Ranges for Protons) reference database [10], the continuous slow-down approximation ranges of 200 MeV and 250 MeV protons in A150 tissue-equivalent plastic are 25.8 cm and 37.69 cm, which are sufficient to penetrate the head and trunk of an adult, respectively [11].

In order to validate the reliability of the GEANT4 simulation results, we compared them with the stopping power values shown in NIST PSTAR data.

**Fig 1:**
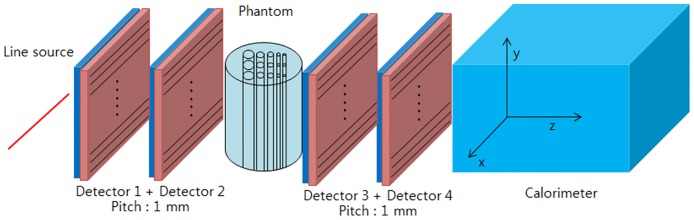
Schematic of pCT configuration. This figure shows the pCT system, which consists of four SSDs and a calorimeter.

**Fig 2:**
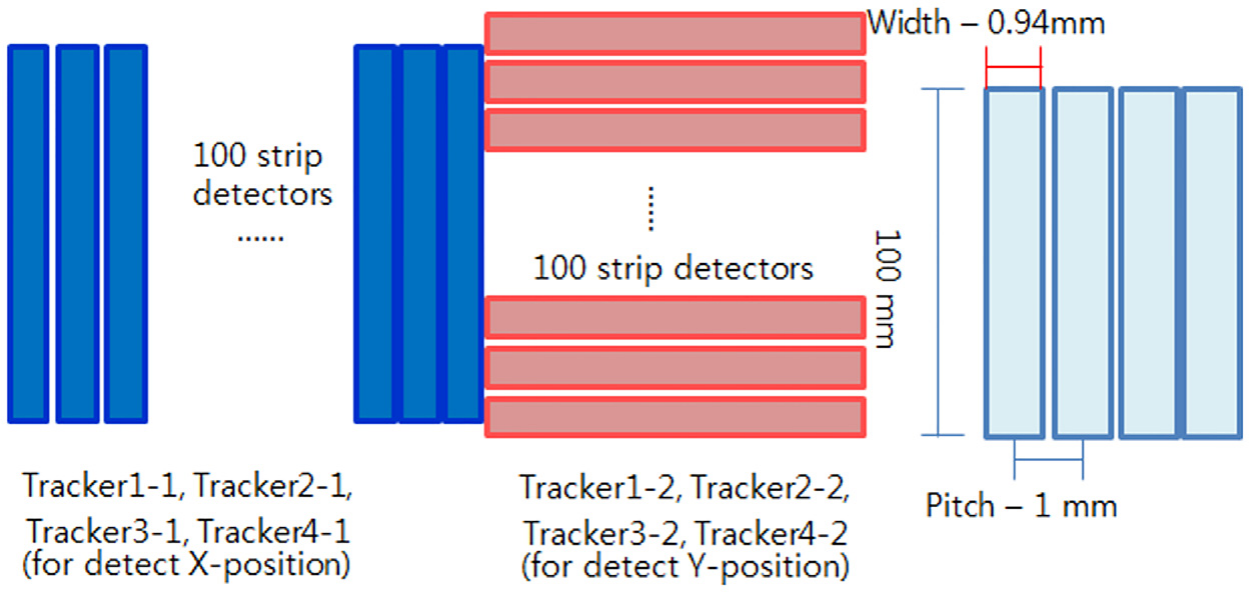
Detailed schematic of tracker geometry. Each single strip in the SSDs has a width of 0.94 mm and a height of 100 mm. The pitch between strips is 1 mm.

The pCT system configuration is shown in Fig 1. A pCT detector is arranged on both sides of the phantom and records the protons’ entrance and exit locations as well as the exit energy *E*_out_ of each proton. The simulated pCT configuration consisted of four silicon strip detectors (SSDs) for proton particle tracking and a calorimeter to measure the residual energy *E*_out_ of each proton. The entrance and exit tracker modules were placed before and after the phantom, respectively, and each tracker consisted of two SSDs. The SSDs were each composed of 100 strips with dimensions of 0.94 × 100 × 0.1 mm^3^ and 100 strips with dimensions of 100 × 0.94 × 0.1 mm^3^. The calorimeter was placed next to the exit module and was composed of cesium iodide crystals with dimensions of 100 × 100 × 200 mm^3^. The detailed tracker geometry and dimension are shown in Fig 2. Single tracker includes 100 strips for X and Y coordinates, respectively. Height of the strip was 100 mm and the width was 0.94 mm. The strip pitch was set by 1 mm.

**Fig 3:**
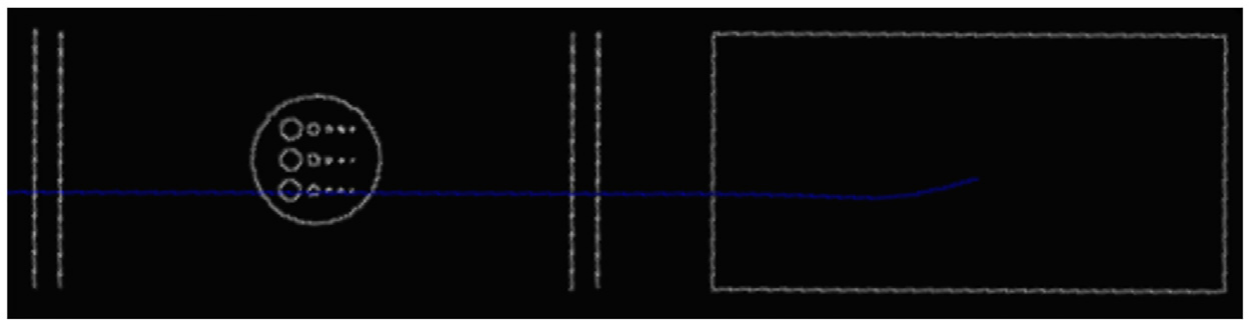
pCT system simulated using GEANT4. This figure shows the GEANT4 pCT simulation.

**Fig 4:**
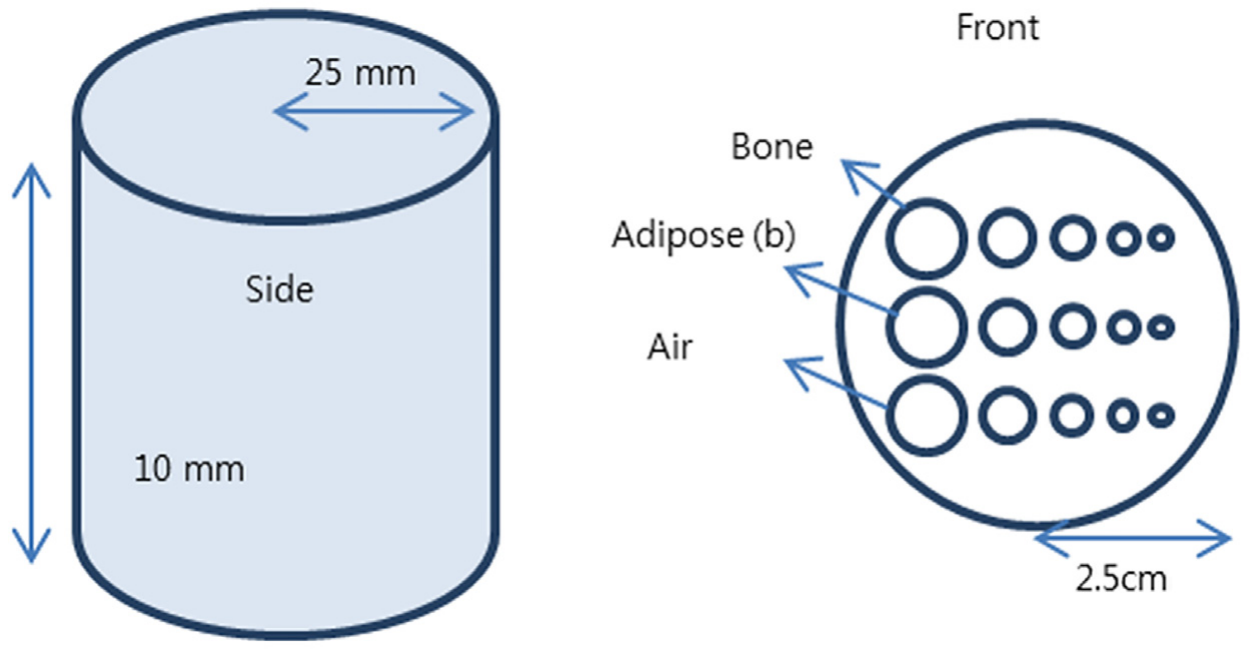
View of phantom. The phantom is composed of bone, adipose, and an air region in a cylinder filled with water.

The pCT system simulated using GEANT4 is shown Fig 3. The phantom was placed between the second and third trackers, and the different projection images were obtained by rotating the phantom. Fig 4 and Table 1 present side and top views and the specifications of the phantom, respectively.

**Table 1:** Hole sizes and materials of phantom.

	Radius	Phantom	Material	Density
Hole 1	4 mm	Upper	Bone	1.85 g/cm^3^
Hole 2	2 mm	Middle	Adipose	0.92 g/cm^3^
Hole 3	1 mm	Lower	Air	0.0012 g/cm^3^
Hole 4	0.75 mm	-	-	-
Hole 5	0.5 mm	-	-	-

### System optimization

To derive ideal pCT design parameters, the effects of the geometrical factors, which were the distances between detectors (specifically, between the first and second detectors and between the third and fourth detectors) and the detector thickness, were estimated. The two factors were the most influential parameters for pCT performance due to the MCS. The spatial resolution of a pCT is physically limited due to protons that are deflected by MCS. The geometrical factors were selected to determine the relations between MCS and the parameter (1.thickness 2. interval distance). The detector thicknesses were 0.01 mm, 0.04 mm, 0.07 mm, 0.1 mm, 0.2 mm, 0.5 mm, 0.8 mm, and 1.0 mm. The intervals between detectors were 10 mm, 30 mm, 50 mm, 70 mm, 100 mm, and 200 mm. To perform the optimization, we examined the MCS and the sensitivity of the pCT detector without the phantom. These processes utilized beams of protons with energies of 200 MeV and 250 MeV, respectively. The output file was organized according to the number of proton signals that penetrated all of the trackers and was sorted to perform image reconstruction. Then, using the optimized geometry, tomographic images were reconstructed by employing three different algorithms (1.FBP, 2.SART, 3.CS).

### Image reconstruction and analysis

In general, proton paths can be estimated by three methods: the straight line path (SLP), cubic spline path, and most likely path [12].

In this study, the SLP method was employed to estimate the proton paths, and the image reconstruction was performed using the Feldkamp–Davis–Kress (FDK) method, SART, and iterative reconstruction with CS regularization. The FDK reconstruction method is analogous to filtered back projection and is conventionally used to reconstruct cone-beam scanners [13].

The SART is based on the algebraic reconstruction technique, which was used to calculate the correction factors in this study, and the concept of the simultaneous iterative reconstruction technique, whose process the algorithm in this investigation followed [14]. Since iterative image reconstruction with CS regularization utilizes CS, complete original data sets can be recovered from under-sampled, sparse data sets [15,16].

In this study, the images were reconstructed using each of these methods after pre-processing the simulation output and were then analyzed and compared with one another. Multiple parameters can be used to evaluate a reconstructed image quantitatively in image analysis. The contrast-to-noise ratio (CNR) is one of the parameters that describes the contrast between a tumor region and the normal tissues surrounding tumor cancer. In this study, ImageJ software was employed to analyze the reconstructed images using CNR. The CNR was derived from the equation

$$CNR=20\times \text{log}\left(\frac{\left|s-b\right|}{\sqrt{\sigma_{S}^{2}+\sigma_{b}^{2}}}\right)dB,$$ (4)

where *s* is the mean value within the region of interest (ROI) and *b* is a mean background noise. *σ*_s_ and *σ*_b_ are standard deviations of *s* and *b*, respectively. The CNR was derived from bone (white region) to water and from air (black regions) to water until third hole's diameter size. Two minor holes were not large enough to set the ROI. Since the density of adipose is practically the same as that of water (0.92 g/cm^3^), the adipose regions in the images were not distinguishable and were excluded from the CNR results. The CNR calculations were repeated for each reconstructed image.

## Results and Discussion

### System optimization

In order to evaluate the reliability of the GEANT4 simulation results, the values obtained for several parameters were compared with the corresponding values provided in the NIST PSTAR database. The simulated stopping powers were also compared with the reference values in this database for identical material conditions [9].

**Fig 5:**
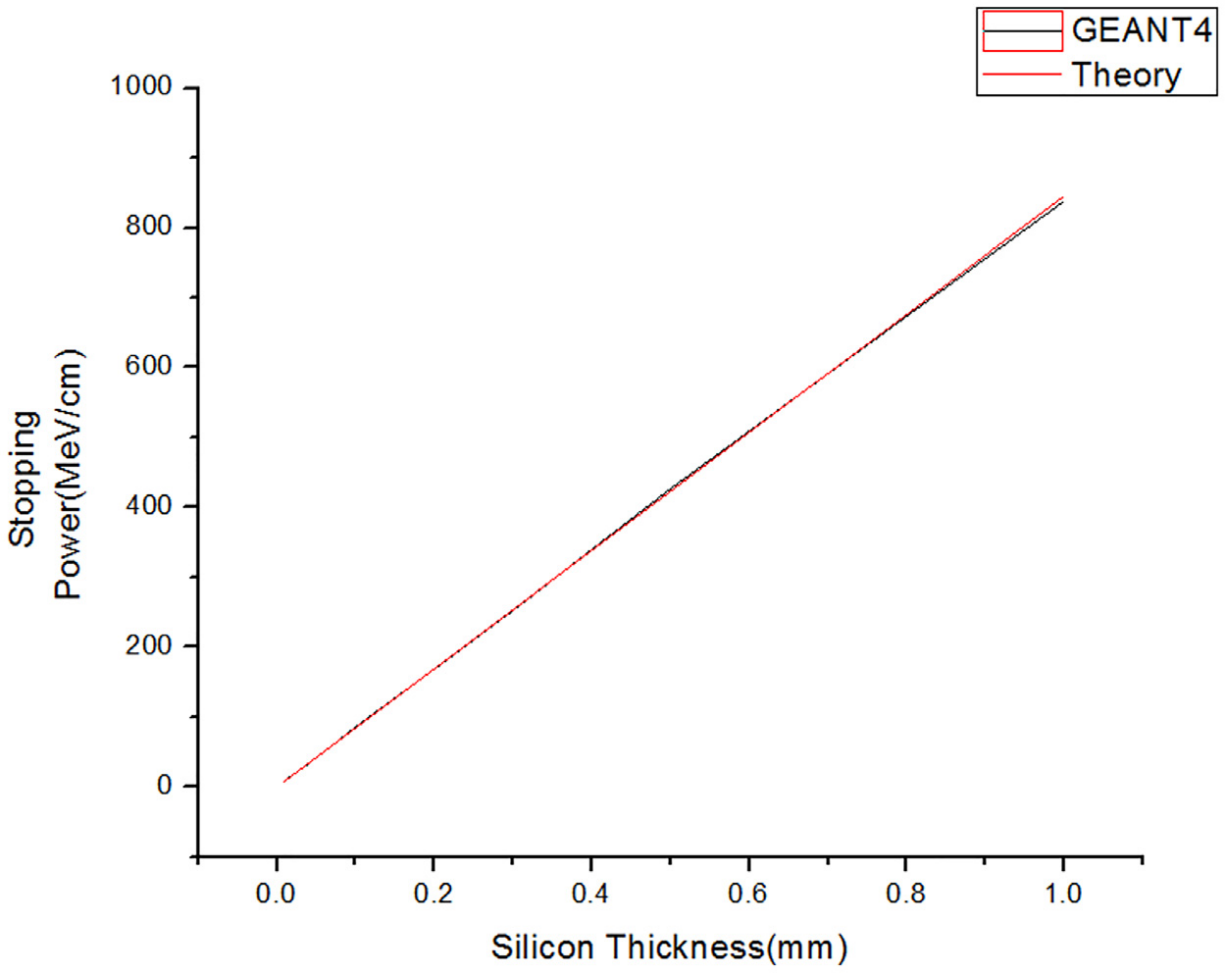
Comparison of GEANT4 results and PSTAR theory. The red line represents the theoretical results from PSTAR, and the black line corresponds to the values obtained from the GEANT4 simulation.

As shown in Fig 5, the stopping power depends upon the strip material thickness in both the GEANT4 simulation results and the NIST PSTAR value. The proton stopping power increases with increasing detector thickness, and the simulated and theoretical results agree well.

**Fig 6:**
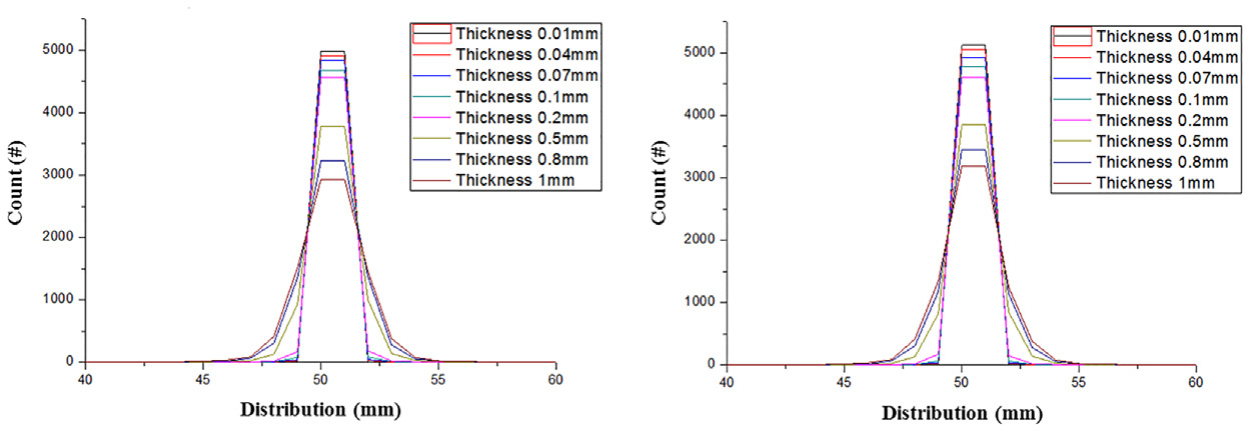
Distributions of 200 MeV (left) and 250 MeV (right) protons for different detector thickness. These graphs depict the beam distributions according to the detector thickness for 200 MeV and 250 MeV protons. The detector thickness was varied from 0.01 mm to 1 mm in the GEANT4 simulation.

Although various factors influence pCT systems, we determined that the tracker thickness and the distance between the entry and exit trackers were the most influential parameters. Therefore, we used these parameters them to optimize the pCT geometry. The effects of varying the thickness were verified by analyzing the proton distributions for different strip thickness, which are shown in Fig 6.

As described above, MCS is a primary cause of image quality degradation, and increased MCS results in greater image inaccuracy.

Tracker thicknesses of 0.01 mm, 0.04 mm, 0.07 mm, 0.1 mm, 0.2 mm, 0.5 mm, 0.8 mm, and 1.0 mm were investigated to determine the optimum thickness that would minimize the MCS. The proton beam was restricted to the Z-axis, and the data obtained by the fourth detector were recorded. The data were recorded along the Y-axis to estimate the proton distributions caused by MCS at the tracker only. In order to neglect the MCS effect in the phantom, the phantom was excluded from the simulation.

Based on the results, it was determined that MCS can occur within a detector and that, as the detector thickness increases, the non-scattered proton count decreases. In addition, the non-scattered proton count distribution becomes narrower with increasing proton energy. This result demonstrates that proton MCS is more influenced by the detector thickness than by the proton energy.

**Fig 7:**
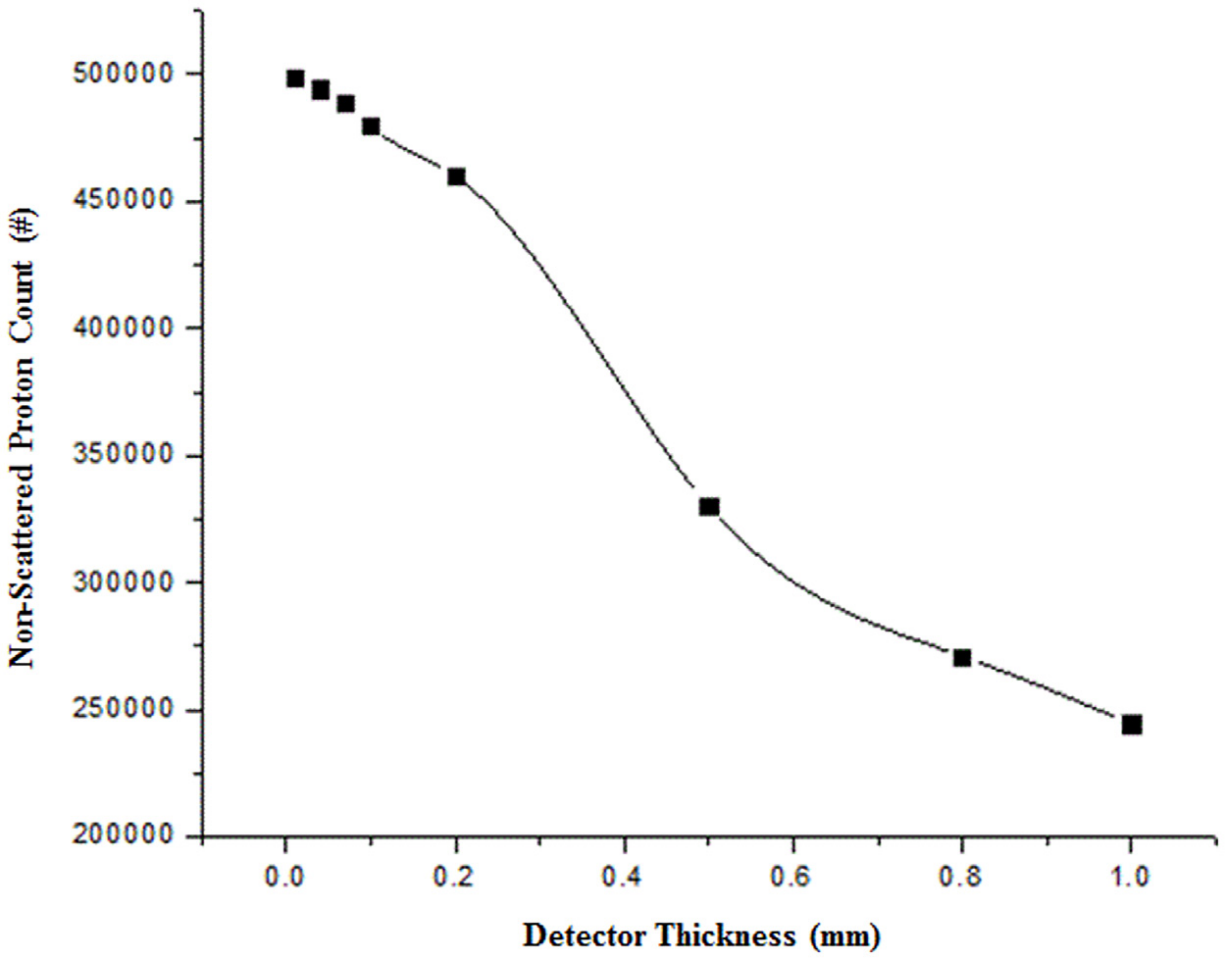
Non-scattered proton counts versus detector thickness. This graph shows the non-scattered proton counts for various detector thicknesses.

Fig 7 shows the number of protons that penetrated all of the trackers without MCS versus the detector thickness. For thicknesses less than 200 μm, the sensitivity variation is relatively slight, but remarkable fluctuations are observable for thicknesses greater than 200 μm. Based on the results, a strip thickness of 200 μm was selected, which could generate sufficiently intense charged particle signals at the electronics and also be simply manufactured. Then, the distance between the detectors was optimized using a fixed strip thickness of 200 μm.

**Fig 8:**
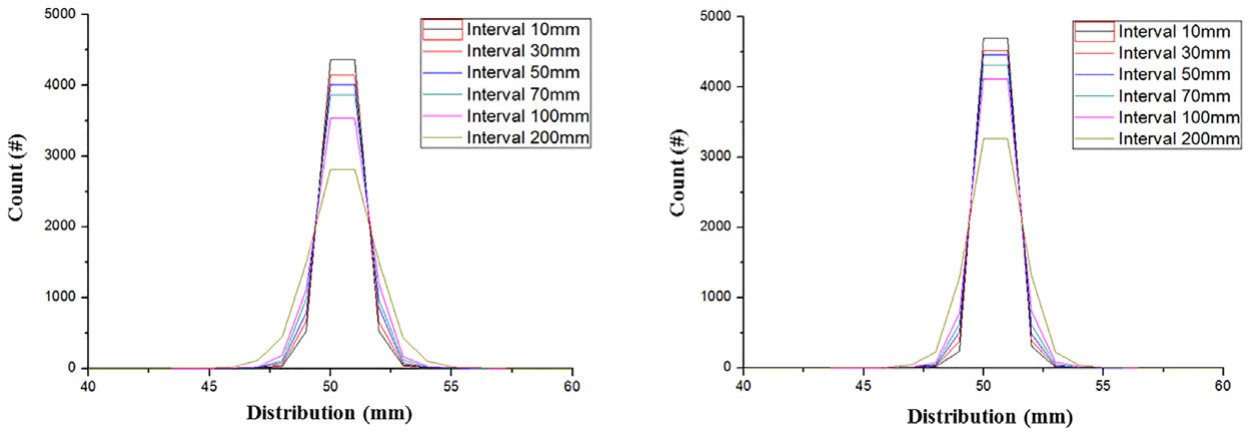
Distributions of 200 MeV (left) and 250 MeV (right) protons for various distances between entry and exit detectors. These graphs depict the beam distributions corresponding to various distances between the detectors for 200 MeV and 250 MeV protons. The distance between the detectors was varied from 10 mm to 200 mm in the GEANT4 simulation.

Fig 8 depicts the beam distributions for various distances between the detectors and 200 MeV and 250 MeV protons. These graphs show that the scattered proton distribution broadens as the distance between the detectors increases. Table 2 lists the numbers of non-scattered 200 MeV and 250 MeV protons counted using various distances between the detectors and reveals that the maximum scattering occurs when the distance is the greatest.

**Table 2:** Number of protons incident on 50^th^ detector.

Interval-scatter-strip-200 MeV and 250 MeV on-50^th^ detector				
interval(mm)	Count (#)		Count (%)	
	200 MeV	250 MeV	200 MeV	250 MeV
10	435,771	465,582	43.57	46.55
30	420,134	453,789	42.01	45.37
50	401,860	440,781	40.18	44.07
70	384,224	427,086	38.42	42.70
100	358,930	405,828	35.89	40.58
200	287,515	336,577	28.75	33.65

In addition, the amount of scattering is lower when the proton energy is greater because MCS is primarily affects low-energy particles. Through the process described above, the optimal distance between the detectors was determined by 10 mm. It was determined that a 200 μm detector thickness and 10 mm distance between the detectors were ideal for this pCT system. The simulation and projection data employed for image reconstruction were then obtained using the optimized system.

### pCT reconstruction results

**Fig 9:**
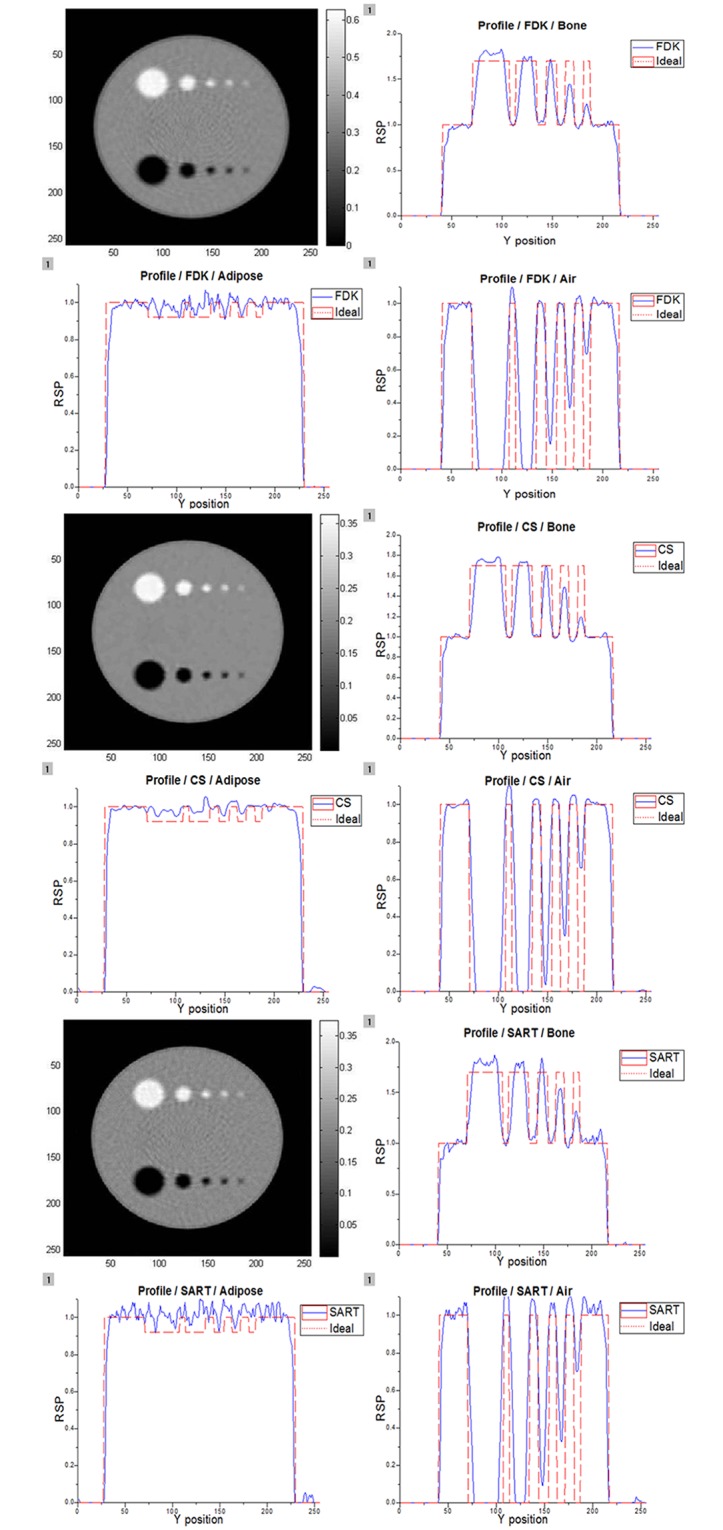
Reconstructed images and profiles obtained using 200 MeV proton beam. These images were reconstructed by three different techniques. The RSP profiles were obtained using Eq 3 to compare with the ideal RSP profile for 200 MeV protons.

**Fig 10:**
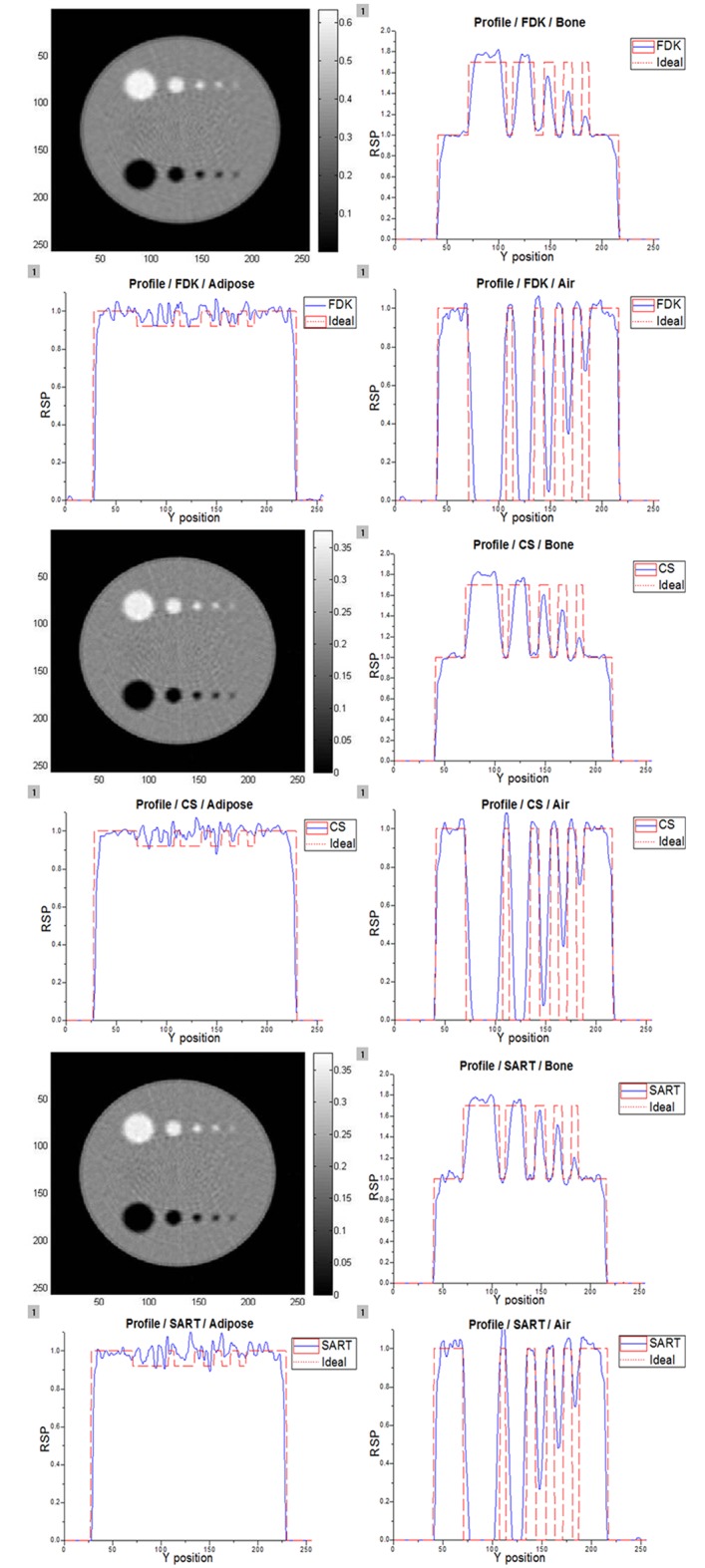
Reconstructed images and profiles obtained using 250 MeV proton beam. These images were reconstructed by three different techniques. The RSP profiles were obtained using Eq 3 to compare with the ideal RSP profile for 250 MeV protons.

In order to determine the most accurate image reconstruction method, the FDK technique, SART, and iterative image reconstruction method with CS regularization were applied to the projection data obtained using 200 MeV and 250 MeV proton beams. The projections were measured in 2° steps, and each projection contained the information from 10^5^ protons. The phantom included bone, adipose, and air holes, and the phantom body was filled with water. Figs 9 and 10 present the images reconstructed using 200 MeV and 250 MeV protons, respectively. In these figures, air and bone are distinguishable, while adipose is not. Adipose has a density similar to that of water; therefore, it cannot be easily differentiated from water. However, a tumor in a pCT image would have a higher density than the surrounding normal tissues. Therefore, the fact that adipose regions cannot be differentiated is insignificant in pCT image analysis. The image profiles were also analyzed to investigate the abilities of the three methods to discriminate between materials. It is evident that air and bone are clearly distinguishable in both the images and the profiles. An ideal RSP curve is also plotted in each graph in Figs 9 and 10 for comparison with the profiles obtained using the different reconstruction methods. In order to estimate the reconstructed images, their CNRs were calculated using Eq 4. The results are presented in Table 3, in which the CS algorithm shows superior performance compared with the others.

**Table 3:** CNR results.

200 MeVCNR values	W^(a)^1	W2	W3	250 MeVCNR values	W1	W2	W3
	B^(b)^1	B2	B3		B1	B2	B3
FDK	25.96	23.19	11.36	FDK	24.48	23.17	13.14
	29.04	27.97	13.29		26.84	27.54	14.75
SART	23.69	26.23	11.70	SART	23.16	22.89	11.25
	27.39	30	13.13		26.47	30.05	13.01
**CS**	**29.58**	**27.45**	**13.23**	**CS**	**27.82**	**23.54**	**16.03**
	**35.41**	**32.86**	**14.64**		**32.56**	**32.66**	**15.28**

^(a)^: CNR value of white region (bone to water) in Figs 9 and 10

^(b)^: CNR value of black region (air to water) in Figs 9 and 10

## Conclusion

In this study, we investigated pCT scanner system optimization. In order to track individual protons, four proton trackers were employed, and a calorimeter was used to measure the individual residual proton energy. The optimization was performed by conducting GEANT4 simulations, and among the multiple parameters, the detector thickness and the distance between the entry and exit detectors were selected as the quantities to be optimized. It was determined that a 200 μm detector thickness and 10 mm distance between the detectors were ideal for this pCT system. With our optimized system, images were reconstructed using beams of 200 MeV and 250 MeV protons, and projection data were obtained in 2° steps, for a total of 180 projections. Each projection contained information from 10^5^ protons.

For image reconstruction, the FDK method, SART, and iterative image reconstruction method with CS regularization were implemented based on SLP estimation. The images obtained using the three methods showed different noise levels; however, the CS method yielded the minimum error with the true RSP value.

This work also demonstrates the potential for performing high-quality pCT during on-line image-guided proton therapy, without increasing the imaging dose. The results show that high-quality pCT images could be reconstructed using 180 evenly sampled proton projections without any streaks or noise, which can appear due to under-sampling or proton starvation.

Finally, our optimized pCT system and several advanced pCT image reconstruction algorithms present that pCT images in which different materials can be distinguished. Further investigations of the detectors and overall system design should be conducted, and actual pCT experiments should be performed based on our optimization results.
